# Dieulafoy's Lesion of the Major Duodenal Papilla Presenting as Obscure Upper Gastrointestinal Bleeding: A Case Report

**DOI:** 10.1002/ccr3.73203

**Published:** 2026-07-27

**Authors:** Aref Arminfar, Soheil Shahramirad, Melika Nasehi, Roya Jahanbazi, Mohammad Danesh Pazhooh, Nasrin Razavianzadeh

**Affiliations:** ^1^ Department of Medical Sciences Sha.C. Islamic Azad University Shahrood Iran; ^2^ Department of Medical Sciences Yazd.C. Islamic Azad University Yazd Iran

**Keywords:** case report, Dieulafoy's lesion, duodenal papilla, endoscopy, gastrointestinal bleeding, rivaroxaban

## Abstract

Dieulafoy's lesion is an uncommon vascular anomaly that can cause life‐threatening gastrointestinal bleeding. While the stomach is the most frequently affected site, involvement of the major duodenal papilla is exceedingly rare and may escape detection with conventional forward‐viewing endoscopy. We report a rare case of papillary Dieulafoy's lesion presenting as obscure upper gastrointestinal bleeding in a patient receiving rivaroxaban. A 69‐year‐old woman with deep vein thrombosis receiving rivaroxaban, hypertension, and hypothyroidism presented with recurrent melena and severe anemia (hemoglobin 5.8–7.1 g/dL). Despite extensive evaluation, including capsule endoscopy, colonoscopy, and two forward‐viewing esophagogastroduodenoscopies, no bleeding source was identified. Persistent anemia and fresh blood in the second portion of the duodenum raised suspicion of a periampullary bleeding source. Side‐viewing duodenoscopy subsequently identified a 1 × 1 mm erythematous lesion with active bleeding at the major duodenal papilla, consistent with a Dieulafoy's lesion. Combination endoscopic therapy with epinephrine injection, bipolar thermal coagulation, and hemoclip placement achieved complete hemostasis. Rivaroxaban was temporarily discontinued, and no recurrent bleeding or post‐procedural pancreatitis occurred during 6 months of follow‐up. This case highlights the diagnostic challenge of papillary Dieulafoy's lesions when conventional forward‐viewing endoscopy is non‐diagnostic. Early side‐viewing duodenoscopy is essential when a periampullary bleeding source is suspected, and combination endoscopic therapy can provide durable hemostasis, even in anticoagulated patients.

## Key Clinical Message

Papillary Dieulafoy's lesion is a rare cause of obscure GI bleeding. When forward‐viewing endoscopy is negative, early side‐viewing duodenoscopy is essential. Combination endoscopic therapy is effective for achieving durable hemostasis, even in anticoagulated patients.

## 1 Introduction

Dieulafoy's lesion (DL) is an important, yet under‐recognized vascular anomaly characterized by a large‐caliber submucosal artery that erodes the overlying mucosa without the presence of a primary ulcer, leading to significant gastrointestinal bleeding [1, 2]. Although first described over a century ago by Georges Dieulafoy, the lesion continues to present diagnostic and therapeutic challenges [3]. The stomach, particularly within 6 cm of the gastroesophageal junction, remains the most common site, while extragastric locations such as the duodenum, esophagus, jejunum, and colon are increasingly reported but remain rare [4, 5]. The incidence of DL accounts for approximately 1%–6% of acute non‐variceal gastrointestinal bleedings [1, 6].

Clinically, Dieulafoy's lesion often presents with recurrent or massive upper gastrointestinal bleeding, manifesting as hematemesis, melena, or hematochezia, and may lead to hemodynamic instability [1, 7]. The condition predominantly affects older adults, with a slight male predominance, and is frequently associated with comorbidities such as cardiovascular disease, diabetes mellitus, and chronic kidney disease [7]. The use of anticoagulant and antiplatelet agents has been implicated as an additional risk factor, potentially exacerbating bleeding severity [7].

Management of DL has evolved considerably over recent decades. While surgical and angiographic interventions were historically preferred, endoscopic therapy has become the mainstay of treatment, providing both diagnostic and therapeutic benefit [6, 8]. Various endoscopic modalities including epinephrine injection, thermal coagulation, and mechanical hemostasis using hemoclips or band ligation have demonstrated high success rates, particularly when applied in combination [6, 8]. The overall mortality rate has markedly declined with the widespread use of early endoscopic intervention [1, 8].

Despite these advances, Dieulafoy's lesion of the major duodenal papilla remains exceptionally rare and poses significant diagnostic difficulties, particularly when standard forward‐viewing endoscopy fails to identify the bleeding source [9, 10]. Dieulafoy's lesion arising from the major duodenal papilla is exceedingly rare and has been described primarily in isolated case reports. This case highlights how a periampullary Dieulafoy's lesion may remain occult despite repeated forward‐viewing and capsule endoscopy, presenting as true obscure gastrointestinal bleeding, and underscores the diagnostic value of early side‐viewing duodenoscopy as well as the safety and effectiveness of combination endoscopic hemostasis in an anticoagulated patient [9, 10].

## 2 Case History/Examination

**FIGURE 1:**
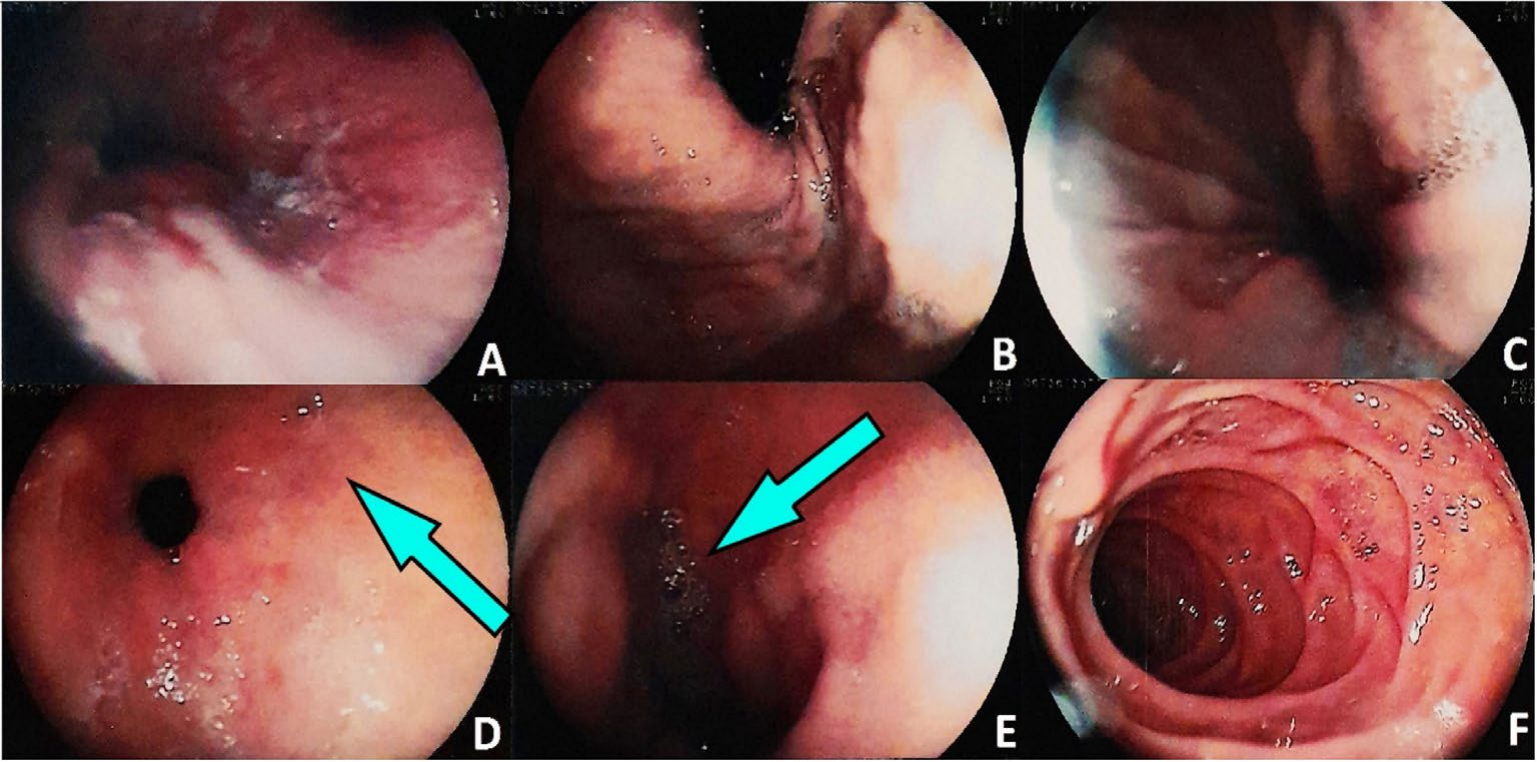
Gastroesophagoduodenoscopy. (A) Lower esophagus with normal mucosal appearance. (B) Gastric fundus with normal mucosal appearance. (C) Gastric body with normal mucosal appearance. (D) Antrum: Mild patchy erythema (blue arrow). (E) Bulb (Blue Arrow). (F) Duodenum 2nd.

A 69‐year‐old Iranian female was referred to our tertiary care for the assessment of recurrent obscure gastrointestinal bleeding, which presented as melena and symptomatic anemia (generalized weakness, dizziness, and exertional dyspnea). Her medical history was significant for hypothyroidism, hypertension, a remote history of coronary angiography, and a previously documented iron deficiency anemia. Prior esophagogastroduodenoscopy (EGD) findings were unremarkable. Findings were conservatively managed with iron supplements which maintained a lower limit of normal hemoglobin level. Two months before the current admission, an outpatient EGD and colonoscopy, peformed as part of the evaluation for anemia, revealed only chronic gastritis (Figure 1). Two weeks prior to the current admission, the patient was hospitalized for a right lower limb deep vein thrombosis (DVT) in the absence of classic risk factors, save for a positive family history, and received overlapping anticoagulation with intravenous Heparin and Rivaroxaban. A subsequent decline in hemoglobin from a baseline of 10.0 g/dL (normal range: 12.0–16.0 g/dL) to 9.4 g/dL (12.0–16.0 g/dL) at discharge was observed, and the patient exhibited signs of rectal bleeding with a positive occult blood (OB) test (normal: negative). Eventually, she was discharged on a therapeutic regimen of Rivaroxaban 15 mg twice daily and recommendations for outpatient follow‐up. Post‐discharge laboratory testing revealed a hemoglobin level of 7.1 g/dL (12.0–16.0 g/dL), prompting the current admission.

On examination, the patient was alert and mildly pale. Her vital signs were within normal limits, demonstrating hemodynamic stability: blood pressure 115/70 mmHg (normal systolic: 90–120 mmHg, diastolic: 60–80 mmHg), heart rate 81 bpm (60–100 bpm), respiratory rate 18/min (12–20/min), temperature 36.6°C (36.1°C–37.2°C) and an oxygen saturation of 98% in room air (≥ 95%). Cardiopulmonary and abdominal examinations were unremarkable.

## 3 Differential Diagnosis, Investigations, and Treatment

During the current admission, she was transfused with 2 units of pRBCs (packed Red Blood Cells) with subsequent clinical improvement. Due to the persistence of anemia and positive OB test (normal: negative) despite a normal EGD, video capsule endoscopy was performed to investigate the small intestine as a potential site of her obscure gastrointestinal bleeding; however, no bleeding source was identified.

The lesion was likely missed on forward‐viewing EGD because of its minute size, flat morphology, intermittent bleeding, and the limited tangential visualization of the papillary region with a standard gastroscope. The key cue prompting side‐viewing duodenoscopy was the repeated observation of fresh blood in the second portion of the duodenum without an identifiable mucosal source, raising suspicion for periampullary origin.

**FIGURE 2:**
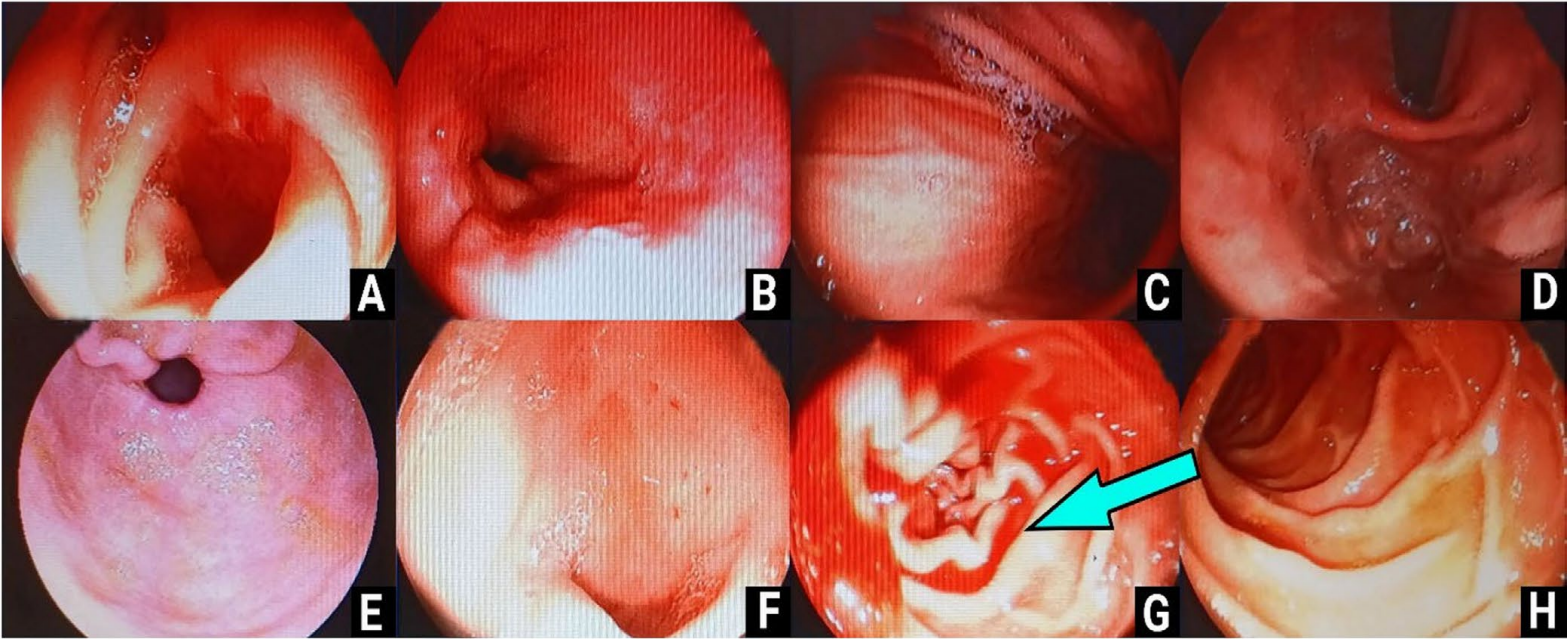
EGD. (A) D1–D2 junction shows a small erosion. (B) Lower esophagus with normal mucosal appearance. (C) Lower esophagus with air bubbles partially obscuring the lumen. (D) Gastric fundus with air bubbles. (E) Antrum: Mild patchy erythema. (F) Duodenum 1st. (G) Duodenum 2nd: Fresh blood was observed (blue arrow). (H) Duodenum 3rd.

Four months after the initial admission, she presented to our facility with a recurrence of melena and severe anemia (hemoglobin 5.8 g/dL [12.0–16.0 g/dL]). She was transfused with 3 units of pRBCs (hemoglobin trend detailed in diagram 2). A repeat EGD was performed, revealing small erosions in the duodenal bulb (D1) and the second part of the duodenum (D2). Fresh blood was noted in D2, raising suspicion for an active bleed from an erosion or ulcer near the papilla and the possibility of hemobilia. Given her rivaroxaban therapy, increased mucosal bleeding related to endoscopic trauma was also considered (Figure 2). Consequently, the patient was considered a suitable candidate for side‐viewing endoscopy to facilitate a definitive evaluation. As side‐viewing endoscopy capabilities were not available at our institution, the patient was referred to a specialized tertiary care center for definitive diagnostic evaluation and management.

Six days post‐discharge, prior to the scheduled side‐viewing duodenoscopy, the patient presented again with hematochezia, dizziness, and retrosternal chest pain (without typical anginal features). Her vital signs remained stable. Cardiac workup, including electrocardiogram (ECG) and qualitative troponin assay (normal: negative), was unremarkable. Laboratory evaluation revealed hemoglobin 7.6 g/dL (12.0–16.0 g/dL). She stabilized with two additional units of pRBCs, achieving a hemoglobin of 9.5 g/dL (12.0–16.0 g/dL) and was subsequently transferred for urgent side‐viewing duodenoscopy.

The definitive side‐viewing duodenoscopy showed a small (1 × 1 mm), erythematous, flat lesion on the major duodenal papilla. During close observation, active fresh bleeding was noted. On side‐viewing duodenoscopy, careful irrigation and close inspection demonstrated active oozing directly from a tiny erythematous lesion on the major papilla, with no alternative bleeding source identified in the adjacent duodenal mucosa. The bleeding was reproducible on observation and localized to the papillary lesion.

Hemostasis was achieved using combination therapy: diluted epinephrine injection (total 10 mL of 1:1000 epinephrine) delivered in small aliquots around the lesion to reduce active bleeding and improve visualization, followed by bipolar thermal coagulation (applied with short pulses using standard settings), and finally mechanical hemostasis with through‐the‐scope hemoclips 2 placed to secure the vessel. Combination therapy was chosen to maximize immediate hemostasis and reduce rebleeding risk in this anatomically high‐risk papillary location.

**FIGURE 3:**
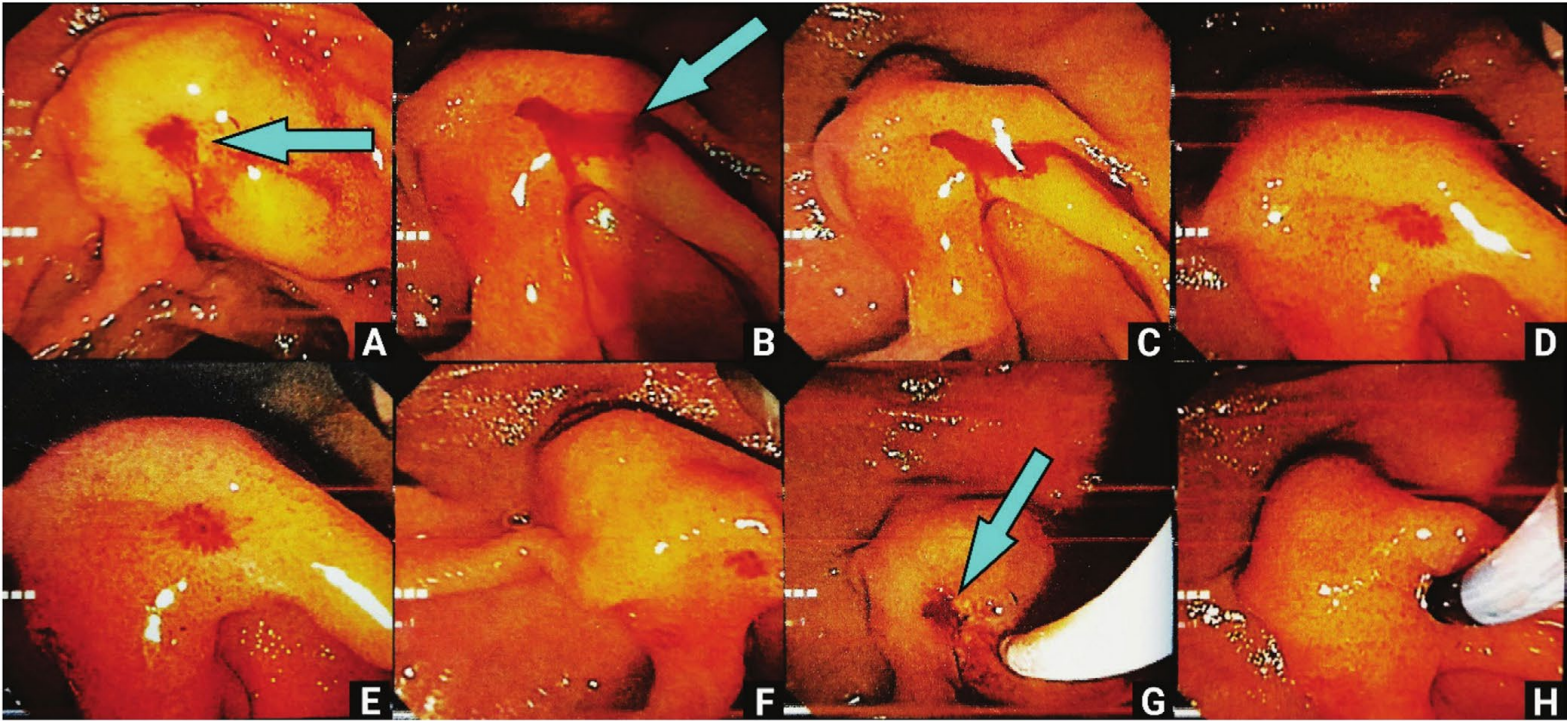
Side‐viewing endoscopic visualization of a Dieulafoy's lesion at the major duodenal papilla. (A) Small erythematous lesion on the major duodenal papilla (blue arrow). (B) Active fresh bleeding from the papillary lesion (blue arrow). (C) Another view confirming ongoing active bleeding. (D) Papillary lesion following epinephrine injection. (E) Reduced bleeding after epinephrine injection. (F) Treated papillary area prior to thermal therapy. (G) Bipolar thermal coagulation applied to the lesion (blue arrow). (H) Successful hemostasis following bipolar thermal therapy.

The final diagnosis was an extra‐gastric Dieulafoy's lesion located on the major duodenal papilla (D2) (Figure 3). This case demonstrates a rare duodenal papillary Dieulafoy's lesion. Its identification requires high endoscopic suspicion and skill. Endoscopic intervention remains the cornerstone of effective diagnosis and treatment, preventing life‐threatening bleeding.

The chronology of the patient's hospital admissions, key findings, and management steps leading to the definitive diagnosis is outlined in Table 1.

**TABLE 1:** Timeline summarizing the diagnostic and therapeutic course of the patient.

Date/time	Clinical event	Key findings	Interventions
2 months before current admission	Outpatient evaluation for anemia	EGD and Colonoscopy: chronic gastritis	No specific treatment; outpatient follow‐up
2 weeks before current admission	Hospitalized for DVT	Doppler ultrasound confirmed DVT	Rivaroxaban 15 mg twice daily
Current admission	Presented with melena and symptomatic anemia (Hb: 7.1 g/dL)	Normal WBC and platelets; INR 2.3; Capsule endoscopy: no bleeding source	Transfused 2 units of pRBC; endoscopy deferred due to rivaroxaban.
4 months later	Recurrent melena and severe anemia (Hb: 5.8 g/dL)	EGD: fresh blood in D2, erosions in D1–D2; MRCP (magnetic resonance cholangiopancreatography): gallbladder sludge ball (possible hemobilia)	Transfused 3 units of pRBC; planned side‐viewing endoscopy.
6 days post‐discharge (final admission)	Presented with hematochezia and dizziness	Hb: 7.6 g/dL; side‐viewing endoscopy: small erythematous papillary lesions with active bleeding. Final diagnosis: Duodenal papillary Dieulafoy's lesion.	Endoscopic injection of Epinephrine and thermal therapy

## 4 Outcome and Follow‐Up

Six months after the procedure, the patient remained symptom‐free with stable hemoglobin; no surveillance endoscopy was performed due to the absence of clinical indications. Rivaroxaban was withheld during this period, and the patient experienced no recurrent melena or hematochezia, stable serial hemoglobin measurements, and no further transfusion or hospitalization. Rivaroxaban was permanently discontinued after successful hemostasis by the treating physician because of concerns regarding the risk of recurrent gastrointestinal bleeding.

This case highlights several important clinical lessons. Papillary Dieulafoy's lesion, although exceedingly rare, should be considered in the differential diagnosis of recurrent obscure gastrointestinal bleeding, particularly when conventional and capsule endoscopies fail to identify a bleeding source. Previous case reports have described similar lesions treated endoscopically, but some were complicated by post‐procedural pancreatitis, likely due to papillary edema or thermal or mechanical injury to the pancreatic duct. In contrast, our patient did not develop pancreatitis, which may be explained by the very small size of the lesion, the targeted and limited application of epinephrine injection and thermal coagulation, and the precise visualization afforded by side‐viewing duodenoscopy. This experience underscores that papillary Dieulafoy's lesions may warrant a high index of suspicion in obscure GI bleeding, that anticoagulation can magnify the clinical severity of otherwise tiny vascular abnormalities, and that careful use of side‐viewing endoscopy with minimally traumatic hemostatic techniques can be both diagnostic and potentially lifesaving while reducing the risk of complications.

## 5 Discussion

Obscure gastrointestinal bleeding remains a major diagnostic challenge in clinical practice. Despite advances in endoscopic and imaging modalities, identifying the bleeding source can be difficult, particularly when the lesion is small, intermittent, or located in an unusual site [11].

Dieulafoy's lesion is a rare but important cause of upper gastrointestinal bleeding, accounting for approximately 1%–2% of all acute cases [10, 12]. Although the lesion most frequently occurs in the stomach, particularly along the lesser curvature, extra‐gastric sites have been reported in the esophagus, duodenum, colon, and rectum [13]. Among these, the duodenum represents only a minority of cases (about 15%), and within this location, involvement of the major duodenal papilla is exceedingly rare, with only sporadic reports in the literature [14].

To the best of our knowledge, this is among the very few reported cases of Dieulafoy's lesion arising from the major duodenal papilla in literature. This highlights the diagnostic challenge associated with small, intermittently bleeding lesions, particularly in uncommon sites such as the major duodenal papilla. Fresh blood in the second part of the duodenum initially raised suspicion of hemobilia or periampullary ulceration.

Alternative causes of periampullary bleeding were considered. Hemobilia was less likely given the absence of biliary colic/jaundice and the lack of endoscopic evidence of blood emanating from the bile duct; similarly, hemosuccus pancreaticus was not supported by clinical or imaging features suggestive of pancreatitis or pancreatic duct bleeding. An ampullary neoplasm was unlikely given the endoscopic appearance of a minute focal vascular lesion without mass effect, ulceration, or irregular tissue. Other vascular malformations were not seen on targeted side‐viewing inspection, and bleeding localized reproducibly to the papillary lesion consistent with a Dieulafoy's lesion.

In our patient, the failure of forward‐viewing conventional endoscopy suggests the critical role of side‐viewing duodenoscopy for optimal visualization and assessment of ampullary or periampullary lesions.

Side‐viewing duodenoscopes provide a tangential view of the papilla, enabling detection of subtle mucosal abnormalities, vascular ectasia, minor bleeding stigmata, or small flat lesions that may be overlooked with standard forward‐viewing scopes. Indeed, guidelines and reviews recommend side‐viewing endoscopy as the modality of choice when evaluating suspected ampullary lesions [15, 16]. In our case, the side‐viewing approach ultimately allowed localization of a tiny (1 × 1 mm) papillary Dieulafoy's lesion and guided definitive endoscopic therapy [17]. This emphasizes that in obscure GI bleeding with suspicion of an ampullary source, side‐viewing duodenoscopy should be considered early in the diagnostic algorithm.

Rivaroxaban, a direct factor Xa inhibitor, has been shown to increase the risk of gastrointestinal bleeding compared with warfarin. In the ROCKET‐AF trial, the incidence of GI bleeding was 3.61 per 100 patient‐years with rivaroxaban versus 2.60 with warfarin [18]. Furthermore, real‐world data suggest that rivaroxaban is associated with a higher rate of GI bleeding than apixaban [19].

In our patient, recent anticoagulation with rivaroxaban likely amplified the severity of bleeding, explaining why an extremely small (1 × 1 mm) papillary Dieulafoy's lesion presented with recurrent melena and profound anemia. It is also important to note that INR is not reliable for monitoring rivaroxaban; rivaroxaban can prolong INR in a dose‐dependent but non‐linear fashion, and the result should not be interpreted as it would be for warfarin [20].

Endoscopic therapy remains the cornerstone of management for Dieulafoy's lesions, with high rates of initial hemostasis and favorable long‐term outcomes. Multiple modalities such as injection therapy, hemoclips, band ligation, and thermal coagulation can be used either alone or in combination. In reported case reports of papillary Dieulafoy's lesions, endoscopic therapy has been successfully applied. For instance, Han et al. described a Dieulafoy's lesion at the major duodenal papilla treated with epinephrine injection and bipolar cautery, achieving successful hemostasis without recurrence [8, 21]. Another case report detailed a 58‐year‐old man with a history of diabetes, hypertension, chronic kidney disease, and chronic calcific pancreatitis presented with five episodes of emesis and melena from a major papilla Dieulafoy's lesion managed with epinephrine injection plus thermocoagulation [21].

In our patient, we achieved definitive hemostasis via combination therapy (epinephrine injection and thermal coagulation) during side‐viewing duodenoscopy, thereby avoiding the need for surgical or radiologic intervention. The success of this approach in prior reports reinforces the strategy used in our case and highlights the importance of timely, skillful endoscopic treatment in such rare and anatomically challenging lesions.

The previously reported cases of papillary Dieulafoy's lesions are summarized and compared with the present case in Table 2, highlighting similarities and differences in clinical presentation, diagnostic methods, therapeutic strategies, and outcomes.

**TABLE 2:** Previously reported papillary Dieulafoy's lesion cases selected and comparison with the present case.

Report	Presentation	Diagnostic method	Endoscopic therapy	Outcome/complications
Inayat et al., 2018 [10]	Melena and anemia	Upper endoscopy	Injection + coagulation	Hemostasis reported
Han et al., 2021 [21]	Emesis and melena	Side‐viewing endoscopy	Epinephrine + bipolar cautery	Hemostasis; no recurrence reported
He et al., 2023 [4]	Massive hematemesis	Endoscopy	Hemoclip	Hemostasis; acute pancreatitis reported
Nakamura et al., 2023 [9]	Obscure GI bleeding	Double‐balloon/side‐viewing endoscopy	Thermal + mechanical therapy	Hemostasis; no recurrence
Present case	Recurrent melena and hematochezia; severe anemia on rivaroxaban	Side‐viewing duodenoscopy after negative workup	Epinephrine + bipolar coagulation + hemoclips	No rebleeding; no pancreatitis at 6 months

## Author Contributions

**Aref Arminfar:** conceptualization, data curation, investigation, methodology, project administration, supervision, writing – original draft, writing – review and editing. **Soheil Shahramirad:** resources, software, validation, visualization, writing – original draft. **Melika Nasehi:** investigation, methodology, resources, software, writing – original draft. **Roya Jahanbazi:** methodology, software, validation, writing – original draft. **Mohammad Danesh Pazhooh:** conceptualization, methodology, supervision, validation. **Nasrin Razavianzadeh:** conceptualization, data curation, project administration, supervision, validation, visualization.

## Funding

The authors have nothing to report.

## Consent

Written informed consent was obtained from the patient for publication of this case report and any accompanying images. A copy of the written consent is available for review by the Editor‐in‐Chief of this journal.

## Conflicts of Interest

The authors declare no conflicts of interest.

## Data Availability

The data that support the findings of this study are available on request from the corresponding author. The data are not publicly available due to privacy or ethical restrictions.
